# Diagnostic Test Analysis of Intraoperative Direct Sonication in Detecting Periprosthetic Joint Infection: A Systematic Review and Meta-Analysis

**DOI:** 10.7759/cureus.110730

**Published:** 2026-06-12

**Authors:** Shunsuke Kondo, Norio Yamamoto, Akihiro Saitsu, Masahiro Ishikane, Yusuke Hirao, Austin Corpuz, Hidehiro Someko

**Affiliations:** 1 Department of Medicine, John A. Burns School of Medicine, Honolulu, USA; 2 Department of Research, Scientific Research Works Peer Support Group (SRWS-PSG), Osaka, JPN; 3 Department of Orthopedic Surgery, Minato Medical Coop-Kyoritsu General Hospital, Nagoya, JPN; 4 R&amp;D Division of Career Education for Medical Professionals, Medical Education Center, Jichi Medical University, Shimotsuke City, JPN; 5 Department of Orthopaedics, Jichi Medical University, Shimotsuke City, JPN; 6 Disease Control and Prevention Center, National Center for Global Health and Medicine, Japan Institute for Health Security, Tokyo, JPN; 7 Department of Internal Medicine, Nagoya Tokushukai General Hospital, Kasugai, JPN; 8 Department of Healthcare Epidemiology, Kyoto University Graduate School of Medicine/Public Health, Kyoto, JPN

**Keywords:** culture media, diagnosis, hip prosthesis, periprosthetic joint infection, sonication

## Abstract

Periprosthetic joint infection (PJI) remains a challenging complication after arthroplasty. Intraoperative direct sonication (sonicating explanted components or periprosthetic tissue in the operating room and inoculating processed fluid into blood culture bottles) may improve pathogen detection by disrupting biofilm. We conducted a systematic review and meta-analysis to evaluate the diagnostic accuracy of this test for suspected PJI. We searched CENTRAL, MEDLINE (PubMed), and Embase (ProQuest) on June 10, 2025, without language restrictions, and screened trial registries (ICTRP, ClinicalTrials.gov), organizational websites/guidelines, citations, and conference abstracts. Eligible studies evaluated intraoperative direct sonication against a predefined reference standard and reported or allowed reconstruction of 2×2 data. Two reviewers independently performed study selection, data extraction, and QUADAS-2 risk-of-bias assessment. We synthesized sensitivity and specificity using hierarchical models. We also generated forest plots and an SROC (summary receiver operating characteristic) curve. The search yielded 1,910 records plus 124 from other sources; six studies met the inclusion criteria. After confirming cohort overlap, four unique datasets remained; two studies (n=479) used the MSIS 2011 criteria and were pooled. Risk of bias was generally low; one study had unclear patient selection. Summary sensitivity and specificity were 88.7% (95% CI, 82.9-92.7) and 83.1% (95% CI, 73.5-89.7), respectively. Intraoperative direct sonication showed promising diagnostic performance for suspected PJI, but the current pooled evidence is based on only two studies using the Musculoskeletal Infection Society (MSIS) reference standard. Larger, multicenter studies using consistent reference standards are needed to refine accuracy estimates and define its role in diagnostic pathways.

## Introduction and background

Periprosthetic joint infection (PJI) is a serious complication in orthopedic surgery, and early identification of the pathogen and appropriate treatment are essential for achieving favorable clinical outcomes [1,2]. One major challenge is biofilm on the implant surface, which protects bacteria and reduces the sensitivity of conventional cultures [3,4]. Since 2006, conventional sonication has been used to process removed implants in sterile containers with dedicated devices to disrupt biofilms and enhance microbial detection, demonstrating a sensitivity of 72-79% and a specificity of 95-97% when evaluated against the Musculoskeletal Infection Society (MSIS) criteria [5-7]. However, this approach requires multiple steps and specialized equipment, making it technically demanding [8]. Additional concerns include contamination during transport, delays in sample processing, and prolonged turnaround time [9]. Several studies have also reported the diagnostic accuracy of conventional sonication methods compared to various gold standards [10-13].

Since 2017, a newer approach, "intraoperative direct sonication," uses a portable ultrasonic device during surgery to sonicate the explanted prosthesis or soft tissue, followed by immediate inoculation into blood culture bottles [7]. This workflow emphasizes portability, the ability to maintain sterility between cases, and compatibility with rapid culture. Within the diagnostic pathway, intraoperative direct sonication has been studied as a triage tool, with several reports showing improved diagnostic accuracy [14-17], and possible indirect cost advantages have been suggested [18].

Recently, several meta-analyses on this topic have been published. However, most existing systematic reviews are limited by small sample sizes and substantial heterogeneity among included studies [19-21]. In previous reviews, intraoperative direct sonication was evaluated together with conventional sonication [22,23]. However, no systematic review or meta-analysis has specifically focused on the diagnostic performance of intraoperative direct sonication alone.

This study aimed to evaluate the diagnostic accuracy of intraoperative direct sonication in patients with suspected PJI.

## Review

Methods and materials

We conducted the review in accordance with the guidance outlined in the Cochrane Handbook and reported it following the PRISMA-DTA (Preferred Reporting Items for Systematic Reviews and Meta-Analyses - Diagnostic Test Accuracy) statement [24] (see Supplemental Material 1). The protocol was registered on the Open Science Framework (https://osf.io/mqang/overview?view_only=77672ec948aa49b3aef95c402e56f6be).

Eligibility criteria for this review

Type of Studies

Studies were eligible if they assessed the diagnostic performance of the index test for PJI using an appropriate reference standard and provided data to derive true positives (TP), false positives (FP), true negatives (TN), and false negatives (FN) values. We included cohort studies, secondary analyses of randomized controlled trials (RCTs), and diagnostic or nested case-control studies. Both published and unpublished work, including conference abstracts and correspondence, were considered without restriction on language or region. Case reports, case series, and studies that lacked sufficient data to construct a 2×2 table were excluded. No limits were placed on follow-up duration.

Participants

We included studies involving patients undergoing diagnostic evaluation for suspected PJI at any infection stage (early postoperative, acute hematogenous, or chronic) and at any joint location (hip, knee, shoulder, elbow, or ankle). Patients with aseptic loosening were also eligible if it was considered within the differential diagnosis. We excluded patients for whom intraoperative sonication could not be conducted or was contraindicated, such as in cases of material hypersensitivity.

Index Test

The index test was intraoperative direct sonication. The contemporary approach to this technique has been outlined in a 2023 report [15]. During surgery, the removed prosthetic components and surrounding tissues were placed separately into sterile 0.9% saline. Sonication was then applied using a handheld device (approximately 25 kHz, pulsed for approximately five minutes), and the resulting fluid was inoculated into paired aerobic and anaerobic blood culture bottles using sterile procedures. For interpretation, we adopted the definitions used in the original studies.

Target Condition

The target condition was PJI, defined as infection involving the prosthetic joint and surrounding tissues [25]. For diagnostic classification, we used the definitions applied in the original studies.

Reference Standard

The reference standard in the included studies was either conventional sonication culture or the MSIS 2011 criteria [26], according to each study’s protocol. Conventional sonication was performed using the approach originally described in 2006 [5]. In brief, explanted prosthetic components were placed in a sterile container, to which 300 mL of 0.9% sodium chloride was added under laminar airflow. The container was vortexed for 30 seconds, sonicated in a 22-L ultrasonic bath for five minutes, and vortexed again for 30 seconds. Ten milliliters of the resulting sonication fluid were then inoculated into paired aerobic and anaerobic blood culture bottles and incubated using an automated blood culture system (e.g., BACT/ALERT 3D).

The MSIS 2011 criteria consider PJI to be present if a sinus tract communicates with the implant or if the same organism is recovered from at least two periprosthetic cultures. Alternatively, PJI is diagnosed when four or more of the following six findings are present: elevated serum inflammatory markers; elevated synovial white cell count; elevated synovial polymorphonuclear proportion; gross purulence; growth of a microorganism from a single periprosthetic specimen; or histological evidence of acute inflammation (≥5 neutrophils per high-power field in at least five fields at ×400).

Outcome

The primary outcomes were sensitivity and specificity. The secondary outcome was the number and proportion of patients who experienced any adverse events. Outcome definitions were taken from those used in the original studies.

Search strategy

Electronic Searches

We searched CENTRAL, MEDLINE (via PubMed), and Embase (via ProQuest) without applying language restrictions (Appendices 1-3). We also searched the WHO International Clinical Trials Registry Platform and ClinicalTrials.gov (Supplemental Materials 4, 5). Additional studies were identified through citation screening of included articles, review of relevant organizational websites and guidelines [26-28], and handsearching of conference abstracts when key primary studies were noted.

Data extraction and analysis

Study Selection

Two of the four reviewers (SK, AS, YH, AC) independently screened titles and abstracts and then evaluated full texts for eligibility. Any disagreements were resolved through discussion or, if needed, adjudication by a third reviewer (NY).

Data Extraction

Two of the four reviewers (SK, AS, YH, AC) independently extracted data, including study location, participant characteristics, sample size, methods used for the index test and reference standard, and 2×2 diagnostic data (TP, FP, TN, FN). Any discrepancies were resolved through discussion or, when needed, by a third reviewer.

We used study-level TP, FP, TN, and FN values for quantitative synthesis. If a study presented multiple results for the same index test, we prioritized them in the following order: external validation dataset, internal test dataset, and then the authors’ main or development dataset. When several estimates were available from the same dataset, we selected the primary prespecified estimate based on this hierarchy. When required, we contacted the corresponding authors for clarification or to obtain missing data.

Assessment of Risk of Bias

Two of the four reviewers (SK, AS, YH, AC) independently evaluated risk of bias and applicability using the Quality Assessment of Diagnostic Accuracy Studies 2 (QUADAS-2) tool [29], adapted for this review (Supplemental Material 6). Disagreements were resolved through discussion, and if consensus could not be reached, a third reviewer was consulted.

Data Analysis and Synthesis

Data analysis followed the recommendations of the Cochrane Handbook. When confusion matrix data were not explicitly reported, we estimated TP, FP, TN, and FN from the total sample size, number of target conditions, sensitivity, specificity, and accuracy (rounded to two decimal places). For studies with zero cells in the 2×2 table, a continuity correction of 0.5 was applied.

Forest plots were generated using a random-effects model and presented in order of sensitivity and specificity. A summary receiver operating characteristic (SROC) curve was used to illustrate the overall distribution of diagnostic performance across studies. We selected the SROC approach because variation in sample volume does not influence incubation results when blood culture bottles are used [30]. A hierarchical model was applied to estimate the SROC curve, assuming variability in diagnostic thresholds across studies.

All analyses were performed using Review Manager version 5.4.1 (Cochrane Collaboration, London, United Kingdom) and R version 4.5.0 (R Foundation for Statistical Computing, Vienna, Austria).

Subgroup Analysis and Sensitivity Analysis

We prespecified subgroup analyses aligned with the primary analysis model. Subgroups included specimen type (synovial fluid, periprosthetic soft tissue, prosthetic surface/sonication fluid), type of arthroplasty (total hip arthroplasty vs. total knee arthroplasty), infection phase (acute vs. chronic), arthroplasty stage (primary vs. revision), PJI episode (first episode vs. recurrent (≥2 episodes)), prior systemic antibiotic exposure (present vs. absent), pathogen Gram classification (Gram-positive vs. Gram-negative), and antimicrobial resistance (resistant organisms, including MRSA (methicillin-resistant *Staphylococcus aureus*), ESBL (extended-spectrum beta-lactamase)-producing, and multidrug-resistant strains vs. non-resistant).

Where sufficient data were available, we conducted sensitivity analyses to examine the stability of results: (1) excluding studies judged to have a high risk of bias in ≥1 QUADAS-2 domain (alternative threshold: high or unclear risk in ≥2 domains), and (2) restricting to studies that used an independent clinical reference standard (e.g., MSIS), excluding those that incorporated sonication culture into the reference standard to limit incorporation bias.

However, because only two studies were included in the quantitative synthesis, statistical tests for heterogeneity, including Cochran’s Q, were considered underpowered and were not used as the sole basis for model selection. We therefore evaluated both fixed-effects and random-effects models as an ad hoc sensitivity analysis. The random-effects model was selected as the primary analysis to account for potential clinical and methodological differences between studies, while fixed-effect estimates were reported as a sensitivity analysis.

Assessment of Heterogeneity

We evaluated heterogeneity by visually reviewing the forest plots of sensitivity and specificity and the SROC plot. We also qualitatively examined potential contributors to heterogeneity, including study design, patient characteristics, variations in the index test, and differences in reference standards.

Assessment of Reporting Bias

To assess publication bias, we searched ClinicalTrials.gov and the WHO ICTRP for completed but unpublished studies. In accordance with the Cochrane Handbook [31], we did not conduct statistical tests for publication bias.

Summary of Findings Table

We assessed the overall certainty of the evidence using the GRADE (Grading of Recommendations, Assessment, Development, and Evaluation) approach [32,33]. For contextual interpretation, we assumed a PJI prevalence of 2% in a general clinical setting [27].

Result

**Figure 1:**
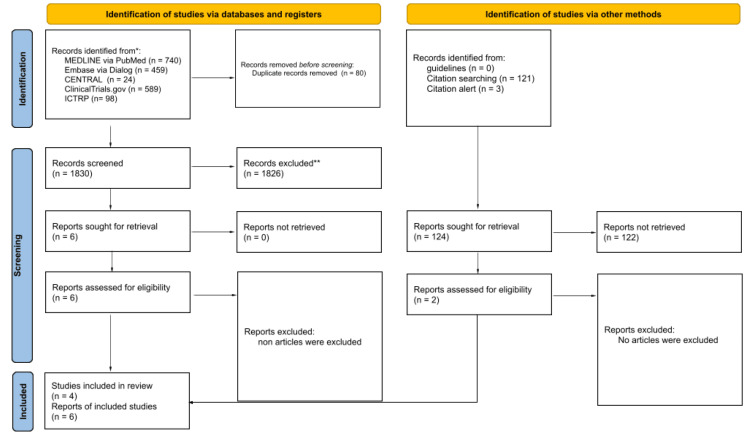
PRISMA 2020 flow diagram for new systematic reviews, which included searches of databases, registers, and other sources *Consider, if feasible to do so, reporting the number of records identified from each database or register searched (rather than the total number across all databases/registers). **If automation tools were used, indicate how many records were excluded by a human and how many were excluded by automation tools. PRSIMA: Preferred Reporting Items for Systematic Reviews and Meta-Analyses

We conducted the systematic search on June 10, 2025, yielding 1,910 records. Citation searching identified 121 additional records, and citation alerts yielded three records. Six reports met the eligibility criteria. Among these, two reports were identified as overlapping with the Li 2025 cohort [34,35], as confirmed by the corresponding author. These overlapping reports were retained in the PRISMA flow diagram and listed in the study characteristics table for transparency but were not included in the quantitative synthesis to avoid double-counting participants. After accounting for cohort overlap, the six eligible reports represented four unique datasets: Padolino 2021 [14], Beguiristain 2023 [16], Ji 2023 [15], and Li 2025 [17]. Two studies using the MSIS 2011 criteria as the reference standard were included in the pooled analysis [14,17]. We retrieved six articles for full-text assessment (six articles from databases and two from alerts), and four studies were included in the review (Figure 1). Full search strategies are listed in Supplemental Material 2.

Table 1 summarizes study characteristics: two prospective studies [15,17] and two retrospective studies [14,16]. Only two studies used the MSIS criteria as the reference standard [15,17]; none used conventional sonication as the reference standard. Thus, 651 patients were represented across the four unique datasets, whereas 479 patients from two MSIS-based studies contributed to the pooled sensitivity and specificity estimates. The risk-of-bias summary is shown in Table 2. One study did not report whether patient sampling was random, resulting in an unclear judgment regarding patient selection in the risk-of-bias and applicability assessments using the QUADAS-2 tool [17]. QADAS-2 signaling questions are listed in Supplemental Material 4.

**Table 1:** Characteristics of the included studies PJI: prosthetic joint infection; MSIS: Musculoskeletal Infection Society; EBJIS: European Bone and Joint Infection Society; CPSI: Committee on Periprosthetic Shoulder Infection; ICM: International Consensus Meeting

Author, year	Country	Type of study	Infected joints	Aseptic joint	Prevalence of infected joint (%)	Methods of the index test	Methods of reference test
Antonio Padolino, 2021 [14]	Italy	Retrospective cohort study	Shoulder	Shoulder	42.4%	Samples were collected in a sterile screw-capped container after changing sterile gloves	CPSI of ICM
Inaki Beguiristain, 2023 [16]	Spain	Prospective cohort study	Not reported	Not reported	33.6%	Transporting prosthetic components explanted during surgery under sterile conditions	EBJIS criteria
Baochao Ji, 2023 [15]	China	Prospective cohort study	Hip 19 (52.8%), Knee 17 (47.2%)	Hip 17(60.7%), Knee 11 (39.3%)	56.3%	Portable handheld ultrasonic cell disruptor device used for direct sonication of implants and soft tissue separately in the operating room	MSIS criteria During surgery
Yicheng Li, 2025 [17]	China	Retrospective cohort study	Hip 143, Knee 121, Elbow 2	Hip 110, Knee 39, Elbow 0	70.6%	Intraoperative direct sonication handheld device	MSIS criteria
Wenbo Mu, 2025 [34]	China	Retrospective cohort study	Not reported	Not reported	Not reported	Intraoperative direct sonication handheld device	Culture of synovial fluid during operation
Tian Haoyang, 2025 [35]	China	Retrospective cohort study	Not reported	Not reported	66.9%	Intraoperative direct sonication handheld device	Conventional culture

**Table 2:** QUADAS-2 tool. Summary of risk of bias and applicability for all included studies

Study	Risk of Bias				Applicability Consensus		
	Patient Selection	Index Test	Reference Standard	Flow and Timing	Patient Selection	Index Test	Reference Standard
Antonio Padolino 2021 [14]	Low	Low	Low	Low	Low	Low	Low
Baochao Ji 2023 [15]	Low	Low	Low	Low	Low	Low	Low
Inaki Beguiristain 2023 [16]	Low	Low	Low	Low	Low	Low	Low
Yicheng Li 2025 [17]	Unclear	Low	Low	Low	Unclear	Low	Low

Primary Outcome

**Figure 2:**
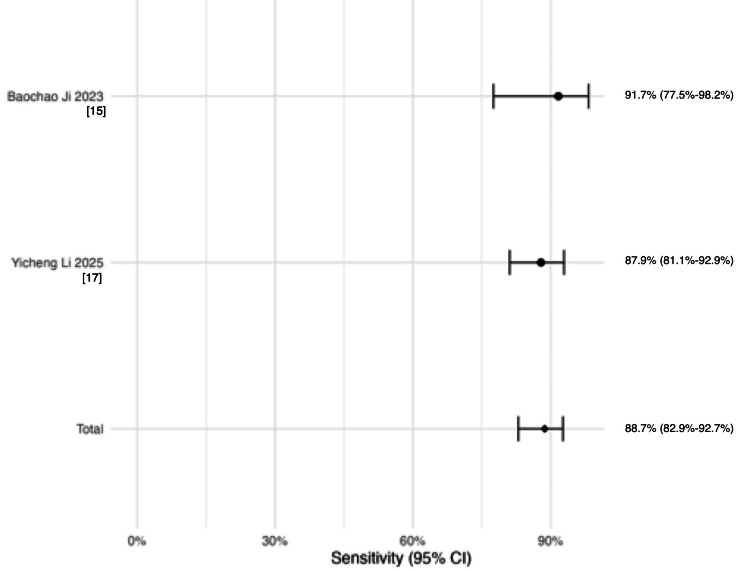
Forest plot sensitivity

**Figure 3:**
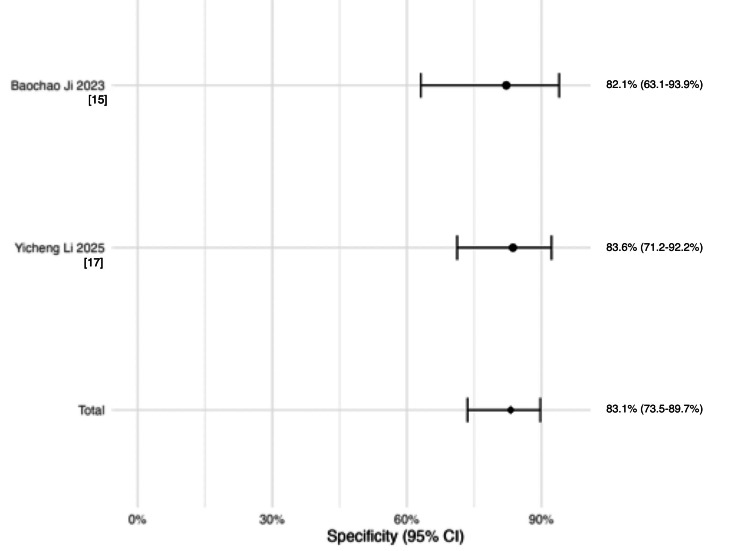
Forest plot specificity

**Figure 4:**
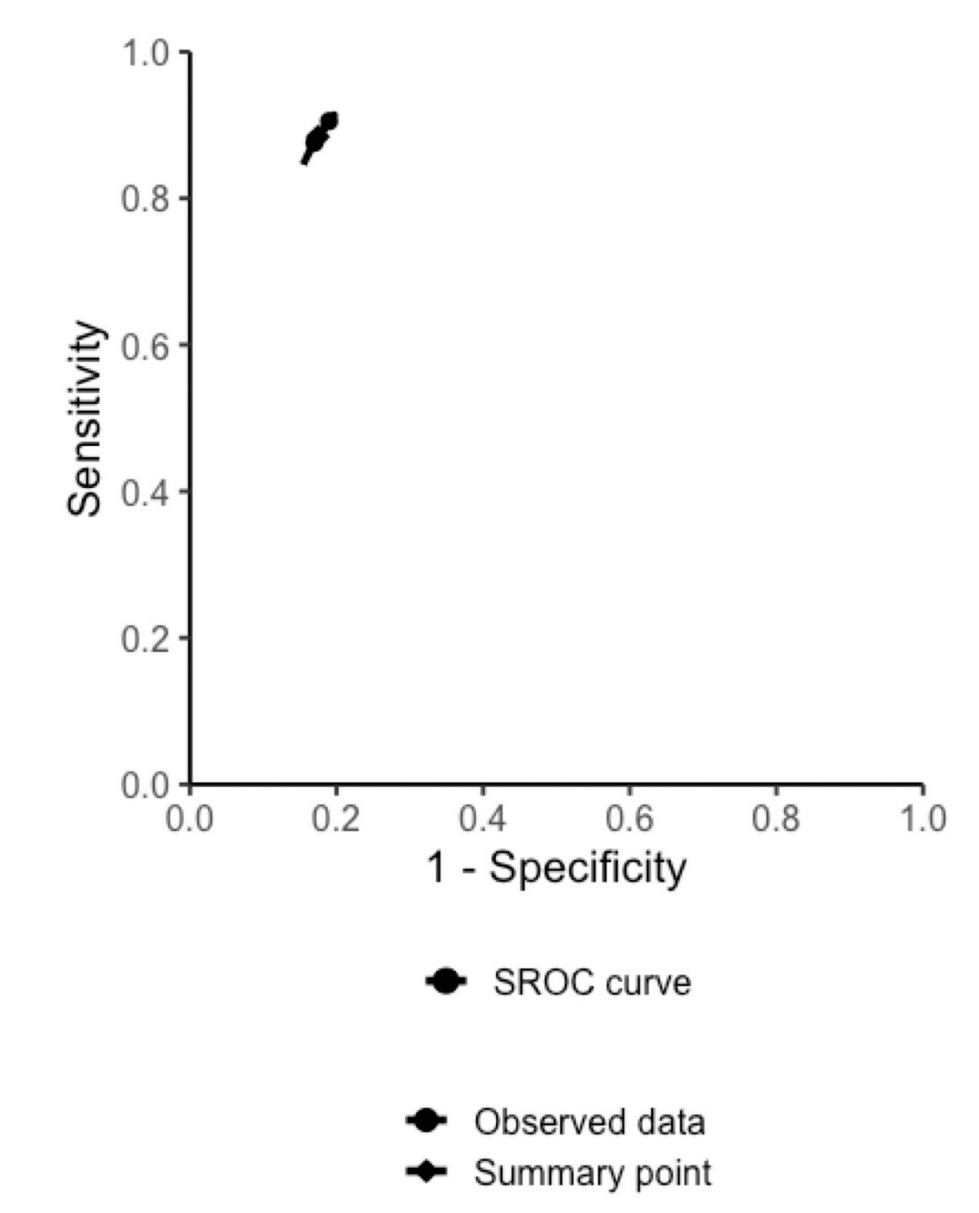
SROC curve SROC: summary receiver operating characteristic

Using studies that applied MSIS as the reference standard, the pooled sensitivity and specificity were 88.7% (95% CI, 82.9-92.7) and 83.1% (95% CI, 73.5-89.7), respectively (Figures 2, 3). The SROC curve is presented in Figure 4. We were unable to report 95% confidence and prediction regions because the number of primary studies was insufficient.

Secondary Outcomes

No study reported adverse events related to intraoperative direct sonication.

Subgroup Analysis and Sensitivity Analysis

Planned subgroup and sensitivity analyses were not performed owing to insufficient data. The sensitivity and specificity according to each reference standard are summarized in Table 3. Across the four unique datasets, the total sample size was 651 patients. However, pooled diagnostic accuracy estimates were calculated only for the two studies that used the MSIS criteria as the reference standard, comprising 479 patients. Therefore, the Summary of Findings table was restricted to these MSIS-based studies and is presented in Table 4. The discrepancy between protocol and manuscript is listed in Supplemental Material 3.

**Table 3:** Summary estimate of direct sonication by each reference standard MSIS: Musculoskeletal Infection Society; EBJIS: European Bone and Joint Infection Society; CPSI: Committee on Periprosthetic Shoulder Infection; ICM: International Consensus Meeting

Index test	Study/Patients, n	Reference test	Sensitivity (95% CI)	Specificity (95% CI)
Intraoperative direct sonication	2 [15,17]/479	MSIS criteria	0.88 (0.82, 0.92)	0.83 (0.73, 0.89)
Intraoperative direct sonication	1 [16]/107	EBJIS criteria	0.69 (0.52, 0.84)	0.94 (0.89, 0.98)
Intraoperative direct sonication	1 [14]/65	CPSI of ICM	0.94 (0.81, 0.98)	0.58 (0.39, 0.75)

**Table 4:** Summary of findings for studies using the MSIS criteria as the reference standard The summary of findings table is based only on the two studies that used the MSIS criteria as the reference standard because pooled estimates were calculated only for this subgroup. The other two unique datasets using EBJIS and CPSI/ICM criteria were summarized separately in Table 3 and were not included in the pooled estimates. MSIS: Musculoskeletal Infection Society; EBJIS: European Bone and Joint Infection Society; CPSI: Committee on Periprosthetic Shoulder Infection; ICM: International Consensus Meeting

Question: What is the diagnostic accuracy of intraoperative direct sonication for prosthetic joint infection in studies using the MSIS criteria as the reference standard?										
Pooled sensitivity: 88.7 (95% CI: 82.9 to 92.7); Pooled specificity: 83.1 (95% CI: 73.5 to 89.7)										
Effect per 1,000 patients tested				Number of patients in pooled MSIS studies (studies)	Factors that may decrease the certainty of evidence					
Test results	Prevalence 1%	Prevalence 2%	Prevalence 3%		Risk of bias	Indirectness	Inconsistency	Imprecision	Publication bias	Certainty of the Evidence (GRADE)
True positives	9 (8, 9)	18 (17, 19)	27 (25, 28)	479 (2)	Low	Not serious	Not serious	Moderate	None	⨁⨁⨁◯Moderate
False negatives	1 (1, 2)	2 (1, 3)	3 (2, 5)							
True negatives	823 (728, 889)	815 (720, 880)	806 (713, 871)	479 (2)	Low	Not serious	Not serious	Moderate	None	⨁⨁⨁◯Moderate
False positives	167 (101, 262)	165 (100, 260)	164 (99, 257)							

Sensitivity Analysis Using Fixed-Effects Model

**Figure 5:**
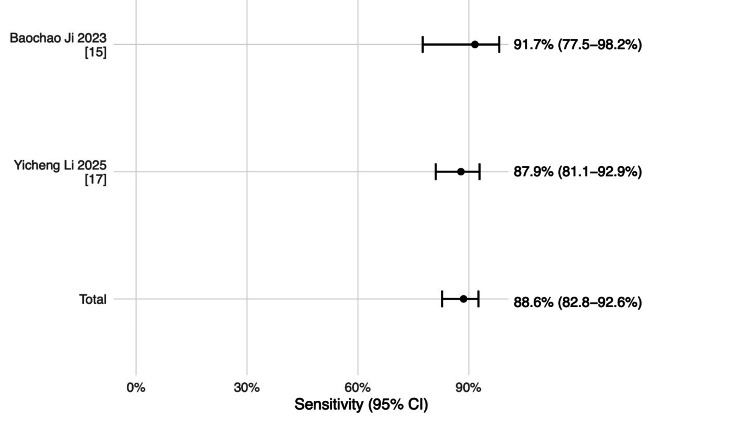
Forest plot sensitivity using fixed-effects

**Figure 6:**
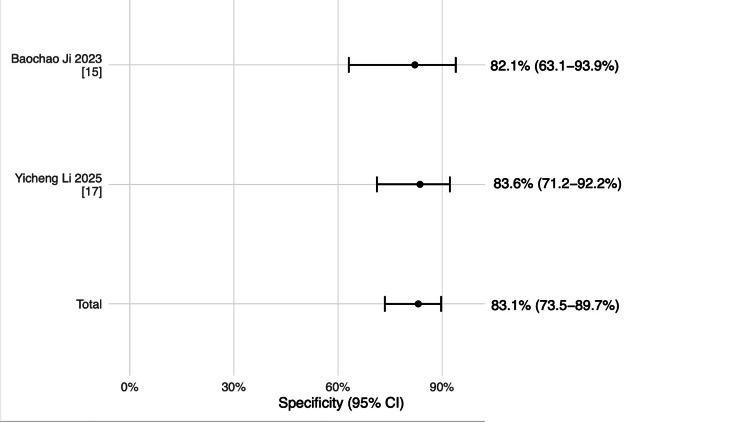
Forest plot specificity using fixed-effects

**Figure 7:**
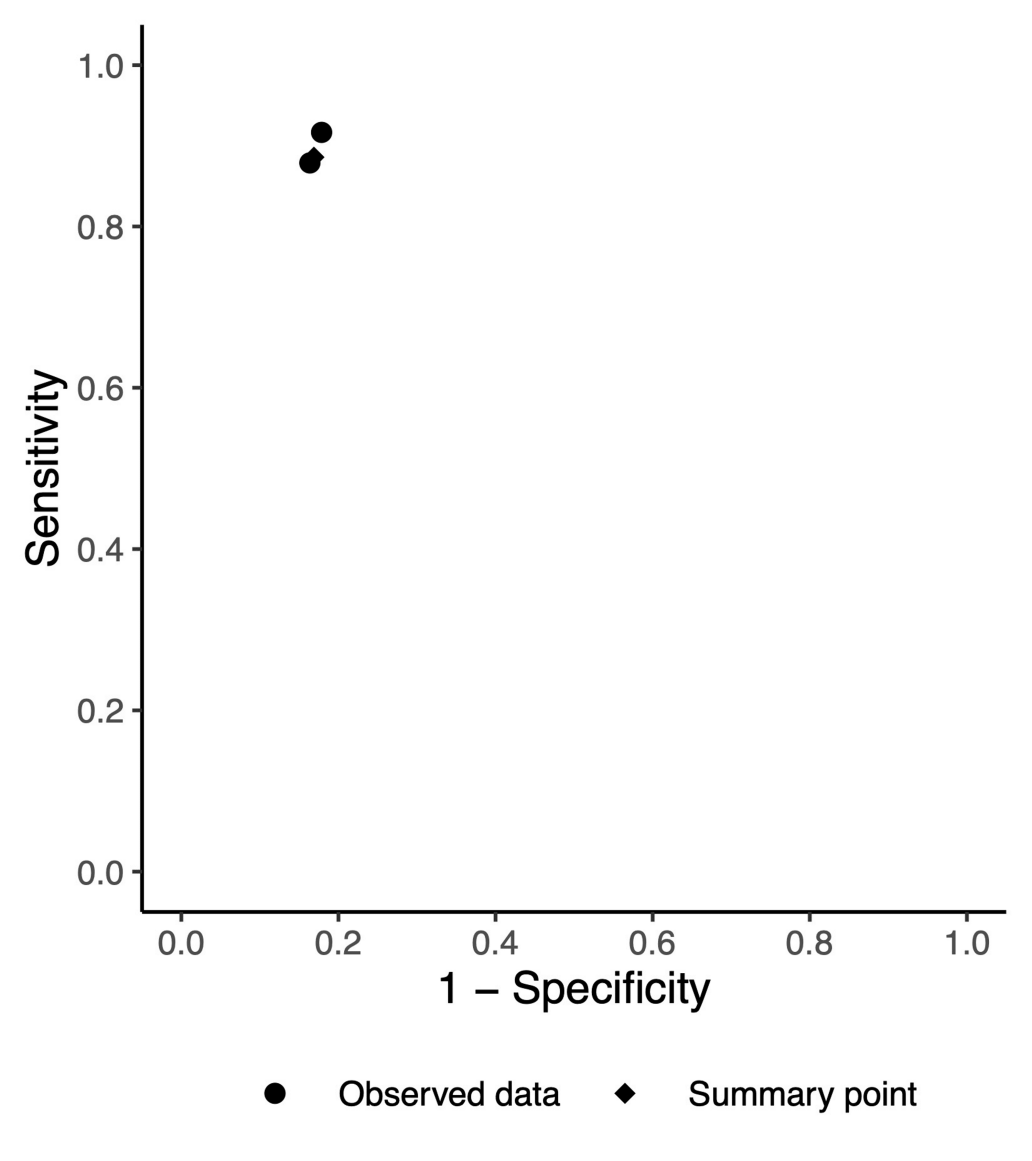
SROC curve using fixed-effects SROC: summary receiver operating characteristic

Using the fixed-effects model, the pooled sensitivity and specificity were 88.6% (95% CI, 82.8-92.6%) and 83.1% (95% CI, 73.5-89.7%), respectively (Figures 5, 6). The SROC curve is presented in Figure 7. These estimates were nearly identical to those obtained using the random-effects model. Thus, the overall interpretation of the diagnostic performance of intraoperative direct sonication was not materially affected by the choice of statistical model.

Discussion

Our review incorporated a total of four studies. QUADAS-2 assessments suggested a low overall risk of bias, with concerns in one study. This systematic review found that, across two studies using MSIS as the reference standard, pooled sensitivity was 88.7% (95% CI, 82.9-92.7) and specificity was 83.1% (95% CI, 73.5-89.7) for the diagnosis of PJI. Adverse events were not reported. Intraoperative direct sonication may be useful to rule out PJI when negative. The pooled estimates were consistent between the random-effects and fixed-effect models, suggesting that the overall interpretation of the diagnostic performance of intraoperative direct sonication was not materially influenced by the choice of statistical model. However, because only two studies were included in the primary quantitative synthesis, statistical measures of between-study heterogeneity should be interpreted cautiously. Therefore, the model choice was based primarily on clinical and methodological considerations rather than statistical heterogeneity testing alone.

Intraoperative direct sonication showed a pooled sensitivity of 88.7% with moderate certainty of evidence (GRADE), suggesting that most true infections would be detected. Because missing a PJI carries substantial consequences, clinicians often prefer sensitivities in the high-90% range for early decision-making [36]. Although intraoperative direct sonication is highly sensitive, it is not superior to all modalities. Histology using permanent sections has been reported to achieve sensitivities >90% [37]. Nevertheless, its applicability may be limited by availability in routine clinical practice, which constrains its widespread use relative to sonication. One practical advantage of intraoperative direct sonication is that specimens can be obtained intraoperatively and sent for incubation immediately after collection. Compared with conventional approaches, sonication also provides an immediate means of disrupting biofilm on prosthetic surfaces without removing the implant, which theoretically enhances microbial yield [15,38]. Taken together, intraoperative sonication offers relatively high sensitivity and may be useful as a triage test.

Current evidence suggests that intraoperative direct sonication is not an adequate replacement for conventional sonication for ruling in PJI. The specificity of intraoperative direct sonication was not high enough to replace conventional sonication, which has a specificity of 95-97% when evaluated against MSIS criteria [5-7]. A plausible explanation is that direct sonication occurs in the operating room: despite aseptic technique and a sterile field, the environment is not microbiologically sterile, and inadvertent co-sonication of native tissue or handling can introduce low-biomass contaminants. In addition, intraoperative direct sonication is performed during surgery, and ultrasonic parameters and subsequent transfer to culture are operator-dependent. This variability may increase false-positive results. In contrast, conventional sonication benefits from standardized processing conditions, including ultrasonic intensity, duration, and post-centrifugation culture protocols, resulting in minimal inter-facility variability. Therefore, clinicians should not use this modality alone to rule in the diagnosis of PJI.

The methodological rigor of this study strengthens confidence in these findings. This review represents the first systematic attempt to pool evidence on intraoperative direct sonication, adhering to PRISMA, the Cochrane Handbook, and the GRADE approach. The comprehensive search, including unpublished protocols, minimized the risk of missing relevant data. In addition, by contacting study authors, we clarified patient overlap across studies and confirmed that individual datasets were unique, thereby reducing duplication bias.

Nevertheless, several limitations should be acknowledged. First, the number of included studies was limited, which reduced statistical power and widened the uncertainty around the pooled estimates. Second, the use of different reference standards limited the ability to conduct uniform meta-analyses, thereby reducing the effective sample size. Third, selection bias and information bias may have influenced individual study results, especially given variation in patient populations and surgical settings. Fourth, the quantitative synthesis was based on only two studies that used the MSIS criteria as the reference standard. This small number of studies substantially limited the precision and robustness of the pooled estimates and prevented the estimation of 95% confidence and prediction regions for the SROC curve. Finally, because the included studies were primarily single-center, generalizability to wider clinical settings remains uncertain. These limitations underscore the need for larger, multicenter investigations to strengthen the evidence base.

## Conclusions

Clinically, intraoperative direct sonication showed promising diagnostic performance for suspected PJI, but the current pooled evidence is based on only two studies using the MSIS reference standard. If the result is negative and the pretest probability is low to moderate, the post-test probability of PJI is likely low. If the result is positive, implant removal should proceed when therapeutically indicated; when composite clinical criteria otherwise establish PJI, conventional sonication may not be necessary and can be reserved for discordant or equivocal cases.

## Appendices

Supplemental material 1

**Table 5:** Preferred Reporting Items for a Systematic Review and Meta-analysis of Diagnostic Test Accuracy Studies (PRISMA-DTA)

Section/Topic	#	PRISMA-DTA Checklist Item	Reported on page #
TITLE / ABSTRACT			
Title	1	Identify the report as a systematic review (+/- meta-analysis) of diagnostic test accuracy (DTA) studies.	1
Abstract	2	Abstract: See PRISMA-DTA for abstracts.	2
INTRODUCTION			
Rationale	3	Describe the rationale for the review in the context of what is already known.	3
Clinical role of index test	D1	State the scientific and clinical background, including the intended use and clinical role of the index test, and if applicable, the rationale for minimally acceptable test accuracy (or minimum difference in accuracy for comparative design).	3
Objectives	4	Provide an explicit statement of question(s) being addressed in terms of participants, index test(s), and target condition(s).	3
METHODS			
Protocol and registration	5	Indicate if a review protocol exists, if and where it can be accessed (e.g., Web address), and, if available, provide registration information including registration number.	3
Eligibility criteria	6	Specify study characteristics (participants, setting, index test(s), reference standard(s), target condition(s), and study design) and report characteristics (e.g., years considered, language, publication status) used as criteria for eligibility, giving rationale.	3
Information sources	7	Describe all information sources (e.g., databases with dates of coverage, contact with study authors to identify additional studies) in the search and date last searched.	4
Search	8	Present full search strategies for all electronic databases and other sources searched, including any limits used, such that they could be repeated.	4
Study selection	9	State the process for selecting studies (i.e., screening, eligibility, included in systematic review, and, if applicable, included in the meta-analysis).	4
Data collection process	10	Describe method of data extraction from reports (e.g., piloted forms, independently, in duplicate) and any processes for obtaining and confirming data from investigators.	4
Definitions for data extraction	11	Provide definitions used in data extraction and classifications of target condition(s), index test(s), reference standard(s) and other characteristics (e.g. study design, clinical setting).	4
Risk of bias and applicability	12	Describe methods used for assessing risk of bias in individual studies and concerns regarding the applicability to the review question.	5
Diagnostic accuracy measures	13	State the principal diagnostic accuracy measure(s) reported (e.g. sensitivity, specificity) and state the unit of assessment (e.g. per-patient, per-lesion).	4
Synthesis of results	14	Describe methods of handling data, combining results of studies and describing variability between studies. This could include, but is not limited to: a) handling of multiple definitions of target condition. b) handling of multiple thresholds of test positivity, c) handling multiple index test readers, d) handling of indeterminate test results, e) grouping and comparing tests, f) handling of different reference standards	5, protocol

Section/Topic	#	PRISMA-DTA Checklist Item	Reported on page #
Meta-analysis	D2	Report the statistical methods used for meta-analyses, if performed.	5
Additional analyses	16	Describe methods of additional analyses (e.g., sensitivity or subgroup analyses, meta-regression), if done, indicating which were pre-specified.	5
RESULTS			
Study selection	17	Provide numbers of studies screened, assessed for eligibility, included in the review (and included in meta-analysis, if applicable) with reasons for exclusions at each stage, ideally with a flow diagram.	6
Study characteristics	18	For each included study provide citations and present key characteristics including: a) participant characteristics (presentation, prior testing), b) clinical setting, c) study design, d) target condition definition, e) index test, f) reference standard, g) sample size, h) funding sources	6
Risk of bias and applicability	19	Present evaluation of risk of bias and concerns regarding applicability for each study.	6
Results of individual studies	20	For each analysis in each study (e.g. unique combination of index test, reference standard, and positivity threshold) report 2x2 data (TP, FP, FN, TN) with estimates of diagnostic accuracy and confidence intervals, ideally with a forest or receiver operator characteristic (ROC) plot.	6
Synthesis of results	21	Describe test accuracy, including variability; if meta-analysis was done, include results and confidence intervals.	6
Additional analysis	23	Give results of additional analyses, if done (e.g., sensitivity or subgroup analyses, meta-regression; analysis of index test: failure rates, proportion of inconclusive results, adverse events).	6
DISCUSSION			
Summary of evidence	24	Summarize the main findings including the strength of evidence.	7
Limitations	25	Discuss limitations from included studies (e.g. risk of bias and concerns regarding applicability) and from the review process (e.g. incomplete retrieval of identified research).	7
Conclusions	26	Provide a general interpretation of the results in the context of other evidence. Discuss implications for future research and clinical practice (e.g. the intended use and clinical role of the index test).	8
FUNDING			
Funding	27	For the systematic review, describe the sources of funding and other support and the role of the funders.	8

Supplemental material 2: search strategies

CENTRAL Search Strategy

(((([mh Sonication]) OR (Sonication:ti,ab)) OR (ultrasonic:ti,ab)) OR (“Arthroplasty, Replacement”:ti,ab)) AND ((((((Prosthesis-Related Infections*) OR (Prosthesis:ti,ab)) OR (“Prosthetic joint infection”:ti,ab)) OR (“Artificial joint infection”:ti,ab)) OR (PJI:ti,ab))

MEDLINE (via PubMed) Search Strategy

((((Sonication[mh]) OR (Sonication[tiab])) OR (ultrasonic[tiab])) OR (Arthroplasty, Replacement[tiab])) AND ((((((Prosthesis-Related Infections*[mh]) OR (Prosthesis-Related Infections*[tiab])) OR (Prosthesis[tiab])) OR (Prosthetic joint infection[tiab])) OR (Artificial joint infection[tiab])) OR (PJI[tiab]))

*EMBASE Search Strategy* 

S1

EMB.EXACT.EXPLODE("Sonication")

S2

AB("Sonication") OR TI("Sonication")

S3

AB("ultrasonic*") OR TI("ultrasonic*")

S4

AB("ultrasonication") OR TI("ultrasonication")

S5

S1 OR S2 OR S3 OR S4

S6

EMB.EXACT.EXPLODE("Prosthesis-Related Infections")

S7

AB("Prosthesis-Related Infections*") OR TI("Prosthesis-Related Infections*")

S8

AB("Prosthesis*") OR TI("Prosthesis*")

S9

AB("Prosthetic joint infection*") OR TI("Prosthetic joint infection*")

S10

AB("Artificial joint infection*") OR TI("Artificial joint infection*")

S11

AB("PJI") OR TI("PJI")

S12

AB("Arthroplasty, Replacement") OR TI("Arthroplasty, Replacement")

S13

AB("joint replacement*") OR TI("joint replacement*")

S14

S6 OR S7 OR S8 OR S9 OR S10 OR S11 OR S12 OR S13

S15

S5 AND S14

ICTRP Search Strategy

Conditions: (Prosthesis-Related Infections* OR Prosthesis OR Prosthetic joint infection OR Artificial joint infection OR PJI)

Intervention: (Sonication OR ultrasonic OR Arthroplasty, Replacement)

ClinicalTrials.gov Search Strategy

(Sonication OR ultrasonic OR Arthroplasty, Replacement) AND (Prosthesis-Related Infections* OR Prosthesis OR Prosthetic joint infection OR Artificial joint infection OR PJI)

Supplemental material 3: discrepancy between protocol and manuscript

We planned to perform subgroup analysis on the following subgroups in the same manner as the primary analysis in the protocol; 

patients grouped by sample origin, comparing sonication fluid obtained from synovial fluid, periprosthetic soft tissue, and prosthetic surface;

patients grouped by type of arthroplasty, total hip arthroplasty (THA) versus total knee arthroplasty (TKA);

patients grouped by infection phase, acute versus chronic periprosthetic joint infection;

patients grouped by arthroplasty stage, primary arthroplasty versus revision (secondary or subsequent) arthroplasty;

patients grouped by PJI episode, first-time PJI versus recurrent PJI (≥2 episodes);

patients grouped by prior antibiotic exposure, presence versus absence of any systemic antibiotic administration;

patients grouped by pathogen Gram stain, Gram-positive organisms versus Gram-negative organisms;

patients grouped by antimicrobial resistance, resistant organisms (including MRSA, ESBL-producing, and multidrug-resistant strains) versus non-resistant organisms;

Also, we planned to perform sensitivity analysis by repeating the analysis to explore the robustness of the results: 

1) by excluding studies that were judged to have a high or unclear RoB in at least four domains in QUADAS-2, 2) by excluding studies that used conventional sonication as a reference test

2) by excluding the studies that did not follow the protocol of intraoperative direct sonication and used conventional sonication as a reference study

Supplemental material 4: QUADAS-2 checklist tailored for this review

Domain 1. Patient Selection

Signaling question 1: Was a consecutive or random sample of patients with suspected PJI enrolled? Signaling question 2: Did the study avoid inappropriate exclusions (e.g., excluding patients eligible for intraoperative direct sonication without clear justification)? Risk of bias: Could the method of patient selection have introduced bias? Concerns regarding applicability: Is there a concern that the included patients (e.g., in terms of PJI phase or site) do not match the review question?

Domain 2. Index Test (Intraoperative Direct Sonication)

Signaling question 1: Were the index test results interpreted without knowledge of the reference standard (blinded interpretation)? Signaling question 2: Were the test procedures (e.g., device frequency, timing, culture inoculation) clearly pre-specified and consistently applied? Risk of bias: Could the conduct or interpretation of intraoperative direct sonication have introduced bias? Concerns regarding applicability: Are there concerns that the execution or interpretation of intraoperative direct sonication in the study differs from the method described in the review protocol?

Domain 3. Reference Standard (Conventional Sonication or MSIS Criteria)

Signaling question 1: Was the intraoperative direct sonication performed consistently using the defined protocol (e.g., device frequency, duration, and saline conditions)? Signaling question 2: Were the criteria for diagnosing PJI (e.g., MSIS 2011 criteria or conventional sonication thresholds) clearly defined and appropriate? Risk of bias: Could the conduct or interpretation of the reference standard have introduced bias? Concerns regarding applicability: Are there concerns that the reference standard (e.g., reliance on MSIS or conventional sonication) does not match the review definition of PJI?

Domain 4. Flow and Timing

Signaling question 1: Was the interval between index test (intraoperative direct sonication) and reference standard sufficiently short (e.g., within the same surgical day) to ensure clinical stability? Signaling question 2: Did all patients receive the same reference standard (MSIS or conventional sonication)? Signaling question 3: Were all enrolled patients included in the final analysis (e.g., no loss due to missing cultures or contamination)? Risk of bias: Could patient flow and timing (e.g., exclusion after enrollment, inconsistent test timing) have introduced bias?
